# Day Admission Surgery Program in a Prospective Payment System: What Are the Financial Incentives?

**DOI:** 10.1177/11786329231222970

**Published:** 2024-01-18

**Authors:** Fabian Grass, Matthias Roth-Kleiner, Nicolas Demartines, Fabio Agri

**Affiliations:** 1 Department of Visceral Surgery, Lausanne University Hospital CHUV, University of Lausanne (UNIL), Lausanne, Switzerland; 2 Medical Direction, Lausanne University Hospital CHUV, University of Lausanne (UNIL), Lausanne, Switzerland; 3 Lausanne University Hospital, Lausanne, Switzerland; 4 Department of Administration and Finance. Lausanne University Hospital, Lausanne, Switzerland

**Keywords:** Day admission surgery (DAS), prospective payment system (PPS), revenue, costs, incentives, financial penalties

## Abstract

**Background:**

Day admission surgery (DAS) is meant to provide a better in-hospital experience for patients and to save costs by reducing the length of stay. However, in a prospective payment system, it may also reduce the reimbursement amount, leading to unintended incentives for hospitals.

**Methods:**

Over a 4-month period in 2021 and based on predefined clinical and logistic criteria, patients from different surgical sub-specialties were identified to follow the institutional DAS program. Revenue-analysis was performed, considering the Swiss diagnosis-related group (SwissDRG) prospective payment policy. Revenue with DAS program was compared to revenue if patients were admitted the day prior surgery (No DAS) using nonparametric pooled bootstrap *t*-test. All other costs considered identical, an estimation of the average cost spared due to the avoidance of pre-operative hospitalization in the DAS setting was carried out using a micro-costing approach.

**Results:**

Overall, 105 inpatients underwent DAS over the study period, totaling a revenue of CHF 1 209 840. Among them, 25 patients (24%) were low outliers due to the day spared from the DAS program and triggering a mean (SD) financial discount of Swiss Francs (CHF) 4192 (2835), yielding a total amount of CHF 105 435. DAS revealed a mean revenue of CHF 7320 (656), compared to CHF 11 510 (1108) if patients were admitted the day before surgery (No DAS, *P* = .007).

**Conclusion:**

In a PPS, anticipation of financial penalties when implementing a DAS for all-comers is key to prevent an imbalance of the hospital equation if no financial criteria are used to select eligible patients. Promptly revising workflow to maintain constant fixed costs for a greater number of patients may be a valuable hedging strategy.

## Introduction

To face increasing healthcare costs in the mid-eighties, a prospective payment system (PPS) replaced the historic cost-based reimbursement system,^1,2^ which was exclusively based on patient’s length of stay (LOS). This strategic change was launched in the US in the mid-eighties and then spread around the world,^3^ included in Switzerland, where a PPS based on disease related groups (DRGs) was adopted since 2012.^4^ The introduction of Diagnosis-Related Groups (DRG) enabled comparison of data across institutions or providers and allowed to assess the performance at different levels of utilization.^5^ The financial risk was transferred from insurers to providers, imposing an incentive to reduce treatment costs.^6^ With the need for cost reduction and benchmarking, new strategies had to be developed to improve efficiency and display competitive advantages.^7,8^ Research focusing on efforts to measure and improve efficiency and value in health care brought many solutions at the disposal of hospitals.^9,10^ An important lever is to enhance patient flow,^11^ achieved through dedicated strategies of flow-enhancing framework^12^ or through more integrated solutions such as enhanced recovery program (ERP).^13^

Historically, all patients scheduled for elective surgery had to be admitted 1 day prior to the operation. Following implementation of ERP, pathways were simplified with standardized clinical and logistic patient care according to evidence-based guidelines.^13^ Implementation of ERP not only led to significantly improved clinical outcomes^14-16^ but promoted the standardization of medical and nursing practices.^17,18^ Continuous monitoring and auditing of clinical practice improved the pathways further to maximize value-adding activities, a concept also known as lean healthcare.^19-21^ In parallel, the burden of ever-increasing healthcare costs was addressed with a more financially-oriented concept of value-based healthcare.^22^

The joint influence of medical innovations and financial pressure led to a dramatic decrease in length of stay (LOS) after elective surgery over the past decades.^23,24^ More recently, in order to further decrease LOS, new tools were deployed like connected mHealth applications.^25,26^ However, ERP pathways and tracking devices focused on postoperative follow-up and recovery, decreasing post-operative LOS. On the other end, day admission surgery (DAS) was implemented to avoid unnecessary and costly pre-operative overnight stays.^27^ This strategy may promote both patient satisfaction and treatment efficiency.

**Figure 1.**
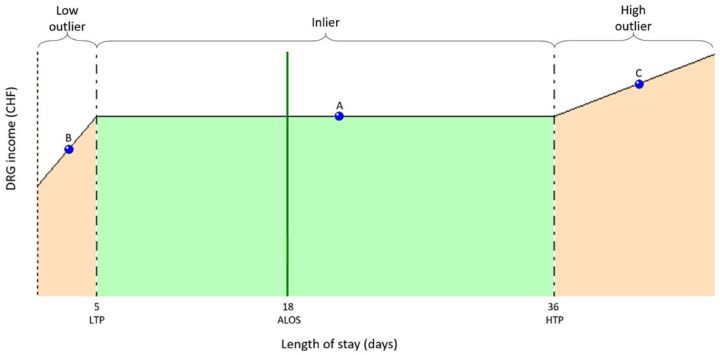
DRG chart: Inlier and outlier reimbursement.

All other services being equal, a shorter stay will reduce the cost per stay. However, shorter stays tend to be more cost intensive due to the resources invested to achieve a prompt hospital discharge.^28^ Also, overly short stays in a PPS could induce adverse effects on the hospital revenue. Indeed, since the Swiss PPS (SwissDRG) ensures fixed fees for stays with statistically defined “normal” LOS for the DRG considered (inliers), outlier LOS for the same DRG trigger a reimbursement amount adjustment. Outliers are patients displaying atypical characteristics relative to other patients in a given DRG. While high LOS outliers trigger a per diem increase in the base reimbursement amount for the hospital, low LOS outliers trigger a financial deduction taken from the base reimbursement amount provided for inliers in that DRG (Figure 1).

It is therefore important to consider low LOS outliers when estimating the financial impact of innovative strategies in a PPS. While extensive literature has analyzed the impact of high LOS outliers on hospitals^6^ and the impact of a PPS on rural critical access hospitals (CAH) in the United States,^29,^30^^ only few studies focused on low LOS outliers in urban acute care hospitals working at maximum capacity most of the time.

The aim of the present study was to analyze the financial impact of DAS implementation for all comers in a tertiary academic hospital with long lasting ERP experience compared to a traditional workflow process where patients are admitted the day before surgery.

## New Contribution

In a PPS, anticipation of financial penalties when implementing new strategies for all-comers is key to promote efficient processes. Authorities in the field should thrive for fair incentives and correct penalties generated by value-added processes. The present work highlights a poor unintended incentive present in the Swiss prospective payment system (SwissDRG) and raises awareness of its impact.

## Methods

Single-center, registry-based retrospective economic evaluation of first consecutive all comers DAS patients in a tertiary academic center between September 1st 2021 and December 31st, 2021. Over this 4-month period, inpatients of 5 different surgical subspecialties, including abdominal surgery, thoracic surgery, cardio-vascular surgery, urology and otorhinolaryngology, followed the newly implemented institutional DAS program with hospital admission at the day of surgery. Patients were offered DAS based on clinical (fit for traveling, no need for further preoperative inpatient investigations or treatments) and logistic (ability to travel in the morning) eligibility criteria. The DAS decision was discussed between surgeons and anesthesiologists. In no case a financial analysis was involved in the decision-making process, and no patient met the criteria for an outpatient strategy.

To monitor the implementation of DAS strategy, a patient register was purposefully created and the present economic evaluation was based on all-comers who benefited from the new strategy during the first 4 months since deployment. Anonymized variables were gathered by querying the hospital’s electronic coding device (Medstat) and included, major diagnostic category (MDC) following the 10th edition of the German modified international classification of disease (ICD-10-GM) 2021 definitions, diagnosis related group (DRG) and its cost-weight, the amount of reimbursement for the hospital (revenue) in Swiss Francs (CHF) and length of stay (LOS).

Revenue analysis was performed with DRG and its linked cost-weight (CW). The actual CW or DAS CW, which determines the amount of the actual hospital revenue, was compared to the simulated DRG’s CW if the same group did not follow a DAS strategy (No DAS). The difference between the actual CW (DAS) and the CW in case of simulated No DAS corresponds to the loss of revenue in the event of low LOS outliers after DAS. The LOS limit at which a case is an outlier is defined for each DRG based on the average LOS of patients falling into that DRG in the previous year. The limit is called *low trim point (LTP)* and corresponds to a third of the average LOS of that DRG but at least 2 days. In the SwissDRG catalog is indicated the first day with reduction, which corresponds to the LTP less 1 day.^31,32^

All other costs being identical between DAS and simulated No DAS groups, an evaluation of the average cost per pre-operative day spared was carried out, using a micro-costing approach.^33^ For this purpose, medical (surgeon and anesthetist visits), nursing and hotel (room and food) costs were used to estimate the average cost related to the preoperative admission day. These costs occur only once within the DAS strategy but twice if the patients are admitted the day before (surgeon and anesthetist visits, nursing) or are completely spared (room and food).

Means (standard deviation SD) and medians (interquartile range IQR) were displayed, depending on the distribution of variables. The nonparametric bootstrap *t*-test with pooled resampling method was used for revenue-analysis.^34^ A *P* value of <.05 was considered statistically significant. Statistical analyses were performed with SPSS_27 (IBM, Armonk, New York, USA) and GraphPad Prism Software 8 (GraphPad Software, Inc., La Jolla, California, USA).

## Results

Over the 4-month period, 105 inpatients underwent DAS. Among the 5 different surgical specialties, the DRGs were related to 12 Major Diagnostic Categories (Table 1).

**Table 1.** Cases by surgical specialties and Major Diagnostic Categories (MDC).

MDC per surgical specialties	N (%)
Urology	31 (30)
Diseases of male genitalia	16
Diseases of urinary organs	15
Abdominal surgery	30 (29)
Diseases of digestive organs	15
Diseases of hepatobiliary and pancreas system	10
Endocrine, nutrition and metabolic diseases	4
Hematologic and solid neoformations	1
Otorhinolaryngology	23 (22)
Diseases of the ear, nose, mound and throat	15
Diseases of the skin, subcutaneous tissue and mammary gland	2
Diseases of the nervous system	2
Hematologic and solid neoformations	2
Endocrine, nutrition and metabolic diseases	1
Injury, poisoning and certain other consequences of external causes	1
Thoracic surgery	19 (18)
Diseases of the respiratory system	13
Hematologic and solid neoformations	3
Diseases of the skin, subcutaneous tissue and mammary gland	2
Injury, poisoning and certain other consequences of external causes	1
Cardiovascular surgery	2 (2)
Diseases of the circulatory system	2
Total	105

The median (IQR) LOS of the 105 patients with DAS was 2 (1-4) days. Among the 105 patients, 25 (24%) were low LOS outliers and all of them stayed only 1 night after surgery.

The median DAS group CW per case was 0.8 (0.6-1.4) point, while the median No DAS group CW per case (only inliers) was 1 (0.7-1.5), corresponding to a relative loss of CHF 53 565 for the actual DAS group compared to the hypothetical No DAS group (Table 2).

**Table 2.** Costs and revenue related to admission strategy.

Costs and revenue	No DAS	DAS
Costs and discounts (CHF)		
Total costs related to the day prior surgery	51 870*	*-*
Total discounts due to low outliers	*-* ^†^	105 *435*
Revenue (CHF)		
Revenue per case (mean, SD)	12 519 (7437)	11 521 (7484)
Total revenue	1 315 275	1 209 840
Net result (total revenue-costs^‡^)	1 263 405	1 209 840
Result (CHF)		
** Delta relative result** ^**^	**+53** **565**	**−53** **565**

* The average cost related to the preoperative admission day was estimated at CHF 494 per patient.

† If all patients were admitted the day before surgery (No DAS), no discount would have been applied.

‡ Assuming all other costs were similar, only the costs related to the day prior surgery are used to compare the actual DAS group to the identical, hypothetical No DAS group.

** The relative gain or loss depending on the strategy (No DAS vs DAS)

The 25 low LOS outliers triggered a 9.9 points of CW reduction, corresponding to a mean (SD) revenue deduction of CHF 4192 (2835) ranging from CHF 1001 to CHF 13 909.

**Figure 2.**
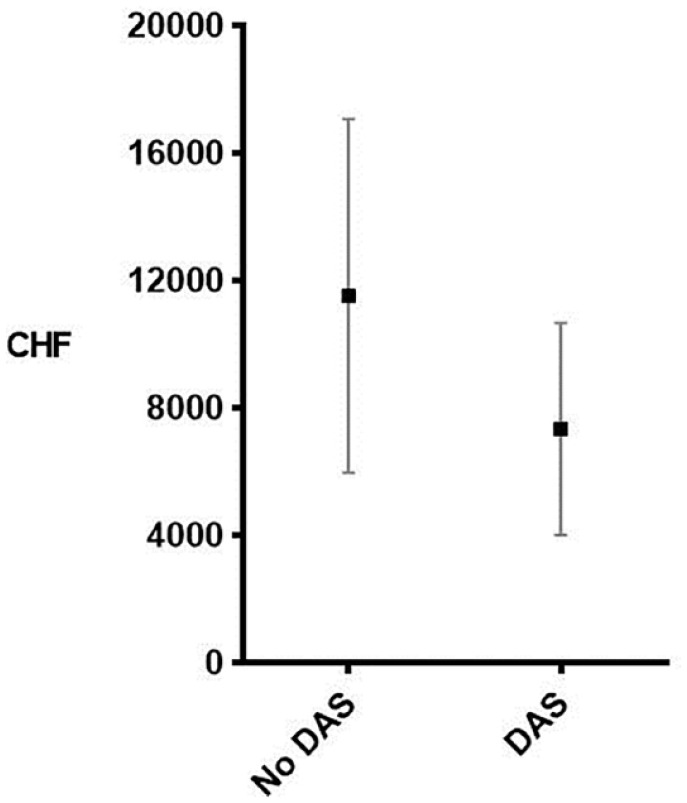
Revenue comparison according to admission strategy.

Hospital reimbursement amount (revenue) comparison of the 25 low LOS outliers with DAS revealed a mean revenue of CHF 7320 (656), compared to CHF 11 510 (1108) if patients were admitted the day before surgery (No DAS, *P* = .007, Figure 2).

## Discussion

The present series displayed a loss of revenue of CHF 105 435 due to 24% low LOS outliers in the DAS group. The same cohort would not have triggered any reimbursement deduction if admitted at the preoperative day (No DAS). The DAS policy did generate some cost savings, corresponding to the preoperative day spared per patient. However, the reimbursement system deducted much more revenue per case due to low outliers. This is a paradox for a health care system thriving toward cost savings and seeking incentives for more efficiency. In a prospective payment system triggering CW adjustment and therefore reimbursement reductions for low LOS outliers, the costs of DAS implementation need to be hedged to prevent a potential imbalance in the hospital equation, thus penalizing innovative hospitals.

## DAS and Low LOS Outliers

While day admission surgery (DAS) became standard in the US, this strategy has not yet been systematically deployed around the world, including in some European countries like Switzerland. The present study reports on the preliminary economic outcome of a newly implemented institutional DAS program, aimed to become a new standard of care. Most countries around the world, including Switzerland, use a prospective payment system (PPS) based on DRGs, with each DRG built on cost-weight calculated annually relaying on updated hospital cost data per case. In the Swiss PPS (SwissDRG), inlier DRGs return to the hospital 100% of the amount determined by the DRG, while low LOS outliers trigger a deduction on the same amount. Eligibility for DAS is fairly based on clinical and logistic criteria. Understanding the possibility of losses of revenue due to SwissDRG financial deduction for low LOS outliers is key to prevent an imbalance of the hospital equation if only clinical and logistic criteria are used to select DAS patients. Low LOS outliers decrease hospital’s financial reimbursement from payers because no patient classification system is able to reliably identify every patient type. Furthermore, the exceptional low LOS outlier patient, representing about less than 5% of all cases,^35^ likely uses less resources than the 95% remaining patients in the same DRG group.^36,37^ Nevertheless, when the reduction is generated solely by an improved strategy (DAS), the incentive results in a penalty for innovative hospitals, threatening the continuation of such innovation.

## Incentives in a PPS

The 1970s marked a period of significant change within the American health care system,^3^ with a sustained impact on health care structures around the world. In September 1976, the journal *Inquiry* published University of Michigan Professor William Dowling’s article “prospective reimbursement of hospitals.”^38^ The new payment system was based on the theory that the cost of medical care was relatively predictable and responsive to changing economic incentives, in particular to lower costs.^39^ At a time where US not-for-profit hospitals presented cost variations of about 100%,^3^ John Thompson at Yale University merged efforts with his colleague Robert Fetter to separate patients into unique “product” categories based on different diagnoses or procedures later called Diagnosis-Related Groups, DRGs.^3,40^ In October 1983, a new hospital-centered payment system was introduced in the United States and continued to evolve and further spread around the world. After its facultative introduction in 2002 in Switzerland, DRG became compulsory in 2012.^41^ As part of the Swiss PPS, outlier payments complement the reimbursement strategy. Case “inliers” for a given DRG have LOS situated between the lower and the upper trim point for a given DRG. High LOS outliers have a longer LOS than the upper trim point and are therefore subject to higher consumption of hospital resources. Low LOS outliers on the other hand, are discharged before the lower trim point and are therefore potentially subject to substantially decreased resource consumption.

**Figure 3.**
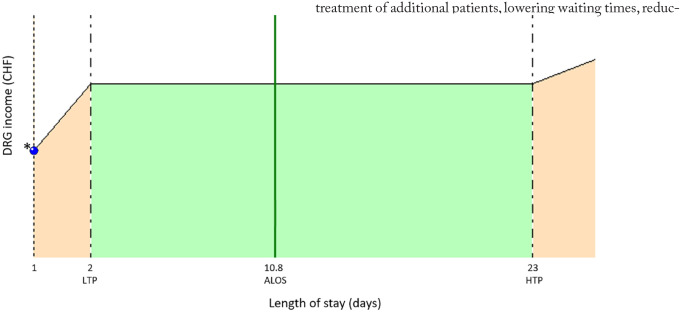
Example of extreme deduction due to a low LOS outlier.

In the present series, the difference that assigned the case to the low LOS outlier category in all situations as a result of the DAS strategy, generated a mean (SD) amount reduction among this group of CHF 4192 (2836), but a loss of revenue of CHF 13 909 in 1 case (Figure 3).

The cost savings related to the avoided preoperative hospital day were estimated at CHF 494 per patient or a total of CHF 51 870 in this present analysis, balancing the overall loss of revenue from CHF 105 435 to CHF 53 565. However, most of these avoided costs are largely fixed (wages and rent) and therefore theoretical.

## Implications

**Figure 4.**
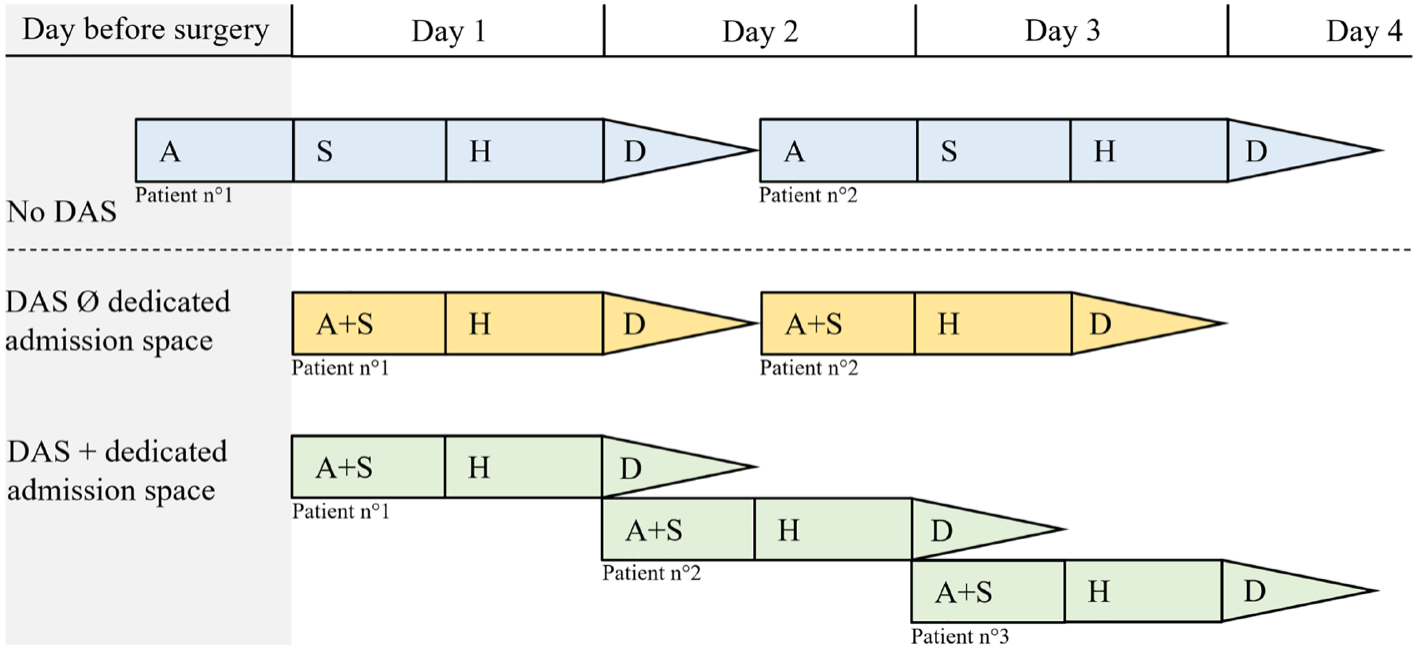
Hedging strategy of day admission surgery (DAS).

At a first glance, the decrease of revenue appears to discourage hospitals to implement DAS. In reality, a DAS program should be considered as long-term investment. First, each DRG has a CW calculated annually on the base of updated hospital cost data per case, shrinking the gap between revenue and costs over time. This mechanism provides a value-based competitiveness to hospitals deploying DAS, while others will anyway face a reduction of their revenue with unchanged costs. Second, the preliminary loss of revenue may be compensated by promptly revising workflow to maintain constant fixed costs for a greater number of patients. This is achievable through logistic optimization including dedicated pre-operative evaluation and admission space.^27^ The patient admitted the same day of surgery does not require an inpatient bed at this stage, but a dedicated reception and preparation area. It is only after leaving the recovery room that the patient will be transferred to a ward bed, available since the previous patient was discharged in the morning (Figure 4). Ultimately, the DAS strategy enables treatment of additional patients, lowering waiting times, reducing opportunity costs, and allowing scale economies.

Furthermore, this strategy encourages an effective use of resources (outpatient pre-operative evaluation, coordination between surgeons, nurses and anesthesiologists), thus reducing unnecessary pre-operative consults and laboratory tests, and decreasing short-term surgery cancelations.^27^ Last but not least, the reduced overall LOS allows for a patient-centered approach, with increased satisfaction,^27,42^ decreased stress and even potentially increased participation in clinical studies.^43^

The suggested hedging mechanisms may help to offer sustainable services with appropriate investments, by diluting the revenue losses caused by low LOS outliers. Furthermore, it is preferable not to invest in a further reduction of the inpatient LOS, but to evaluate a shift to ambulatory care. Otherwise, the Swiss PPS may need to be revised with adjusted trim points for low LOS outliers and creation of dedicated DRGs to help hospitals plan and promote a DAS strategy. This way, hospitals are rewarded instead of penalized for implementing an efficient process. Until then, hospitals should remove the focus from prior financial analyses when deciding on the implementation of a DAS strategy. In the meantime, authorities in the field should thrive for attractive conditions and correct inadequate incentives.

## Limitations

Cost analysis focused on the in hospital setting and did neither take into account outpatient costs, nor costs needed to develop and implement the pre-operative evaluation clinic and related costs. We assumed that the costs were identical between the group treated according to the DAS strategy and the same hypothetical group admitted the day before the intervention. Indeed, this design was chosen because the hospital would have offered the same clinical pathway to the No Das patient apart from hospital admission the day before surgery. The only difference would have been related to additional costs of accommodation and medical care for that supplementary day. Moreover, the low sample size and the short evaluation period may contain uncaptured seasonality patterns. Although these limitations do not change the overall conclusion, a longer observation period is needed to analyze the financial impact on the hospital’s balance sheet.

The present results are representative of the activity of an academic hospital with longstanding ERP experience, which may facilitate the creation of low length of stay outlier cases. Finally, while comparisons in term of cost-weight can be direct between hospitals, the displayed total costs and revenue apply to the present institution and cannot be uncritically extrapolated to other hospitals or settings.

## Conclusion

In a PPS like the SwissDRG, anticipation of financial penalties when implementing DAS for all-comers is key to prevent a negative imbalance if no hedging strategy is planned. Reimbursement amount adjustment following reduced costs related to DAS cases could offer a competitive advantage to hospitals deploying a DAS strategy on the long run. However, authorities in the field should thrive for attractive conditions and quickly correct inadequate financial incentives potentially hindering the deployment of efficient and value-added processes.

## References

[1] Fetter RB Shin Y Freeman JL Averill RF Thompson JD. Case mix definition by diagnosis-related groups. Med Care. 1980;18:iii,1-iii,53. 7188781

[2] Freeman JL Fetter R Nowbold R Rodrigues J. Hospital utilisation before and after the implementation of DRGs for hospital payment: US, 1979–1984. J Manag Med. 1986;1:309-323.

[3] Mayes R. The origins, development, and passage of Medicare’s revolutionary prospective payment system. J Hist Med Allied Sci. 2007;62:21-55. 16467485 10.1093/jhmas/jrj038

[4] SwissDRG. Les forfaits par cas dans les hôpitaux suisses. Information de base pour les professionnels. Accessed December 22, 2022. https://www.swissdrg.org/application/files/4115/0234/7369/170810_SwissDRG_Broschuere_f.PDF

[5] Freeman JL Fetter RB Park H , et al. Diagnosis-related group refinement with diagnosis- and procedure-specific comorbidities and complications. Med Care. 1995;33:806-827. 7637403 10.1097/00005650-199508000-00006

[6] Felder S. The variance of length of stay and the optimal DRG outlier payments. Int J Health Care Finance Econ. 2009;9:279-289. 19107594 10.1007/s10754-008-9051-1

[7] Busse R Geissler A Aaviksoo A , et al. Diagnosis related groups in Europe: moving towards transparency, efficiency, and quality in hospitals? BMJ. 2013;346:f3197. 10.1136/bmj.f3197 23747967

[8] Fetter RB. Diagnosis related groups: understanding hospital performance. Interfaces. 1991;21:6-26.

[9] McGlynn EA. Identifying, categorizing, and evaluating health care efficiency measures. Final Report. Agency for healthcare research and quality; 2008. April (prepared by the Southern Californian Evidence-based Practice Center-RAND Corporation, under contract No. 282-00-005-21). AHRQ Publication No. 08-0030. Accessed October 23, 2023. https://library.ahima.org/PdfView?oid=81708

[10] Fraser I Encinosa W Glied S. Improving efficiency and value in health care: Introduction. Health Serv Res. 2008;43:1781-1786. 18811736 10.1111/j.1475-6773.2008.00904.x PMC2654165

[11] Orszag PR Emanuel EJ. Health care reform and cost control. N Eng J Med. 2010;363:601-603. 10.1056/NEJMp1006571 20554975

[12] Valente R Santori G Stanton L Abraham A Thaha MA. Introducing a structured daily multi-disciplinary board round to safely enhance surgical ward patient flow in the bed shortage era: a quality improvement research report. BMJ Open Qual. 2023;12:e001669. 10.1136/bmjoq-2021-001669 PMC10069591 36972925

[13] Gustafsson UO Scott MJ Hubner M , et al. Guidelines for perioperative care in elective colorectal surgery: enhanced recovery after surgery (ERAS®) society recommendations: 2018. World J Surg. 2019;43:659-695. 30426190 10.1007/s00268-018-4844-y

[14] Noba L Rodgers S Chandler C , et al. Enhanced recovery after surgery (ERAS) reduces hospital costs and improve clinical outcomes in liver surgery: a systematic review and meta-analysis. J Gastrointest Surg. 2020;24:918-932. 31900738 10.1007/s11605-019-04499-0 PMC7165160

[15] Ashok A Niyogi D Ranganathan P , et al. The enhanced recovery after surgery (ERAS) protocol to promote recovery following esophageal cancer resection. Surg Today. 2020;50:323-334. 32048046 10.1007/s00595-020-01956-1 PMC7098920

[16] Pache B Martin D Addor V Demartines N Hübner M. Swiss validation of the enhanced recovery after surgery (ERAS) database. World J Surj. 2021;45:940-945. 10.1007/s00268-020-05926-z PMC7921022 33486583

[17] Hübner M Addor V Slieker J , et al. The impact of an enhanced recovery pathway on nursing workload: a retrospective cohort study. Int J Surg. 2015;24:45-50. 26523495 10.1016/j.ijsu.2015.10.025

[18] Martin D Roulin D Grass F , et al. A multicentre qualitative study assessing implementation of an enhanced recovery after surgery program. Clin Nutr. 2018;37:2172-2177. 29129637 10.1016/j.clnu.2017.10.017

[19] Francis NK Walker T Carter F , et al. Consensus on training and implementation of enhanced recovery after surgery: a Delphi study. World J Surg. 2018;42:1919-1928. 29302724 10.1007/s00268-017-4436-2

[20] Roulin D Muradbegovic M Addor V , et al. Enhanced recovery after elective colorectal surgery - reasons for non-compliance with the protocol. Dig Surg. 2017;34:220-226. 27941313 10.1159/000450685

[21] van Rossum L Aij KH Simons FE van der Eng N ten Have WD. Lean healthcare from a change management perspective: the role of leadership and workforce flexibility in an operating theatre. J Health Organ Manag. 2016;30:475-493. 27119398 10.1108/JHOM-06-2014-0090

[22] Tsevat J Moriates C. Value-based health care meets cost-effectiveness analysis. Ann Intern Med. 2018;169:329-332. 30083766 10.7326/M18-0342

[23] Or Z Häkkinen U . DRGs and quality: For better or for worse? In: Diagnosis-related groups in Europe - moving towards transparency, efficiency and quality in hospitals (eds) Open University Press. Maidenhead: McGraw-Hill House, 2011, pp 115-129.

[24] Pascal L Polazzi S Piriou V , et al. Hospital length of stay reduction over time and patient readmission for severe adverse events following surgery. Ann Surg. 2020;272:105-112. 30676380 10.1097/SLA.0000000000003206

[25] Agri F Hahnloser D Demartines N Hübner M. Gains and limitations of a connected tracking solution in the perioperative follow-up of colorectal surgery patients. Colorectal Dis. 2020;22:959-966. 32012423 10.1111/codi.14998

[26] Agri F Hübner M Demartines N Grass F. Economic considerations of a connected tracking device after colorectal surgery. Br J Surg. 2021;108:e407-e408. 10.1093/bjs/znab377 34738102

[27] Silvay G Goldberg A Gutsche JT T Augoustides JG. Same day admission for elective cardiac surgery: how to improve outcome with satisfaction and decrease expenses. J Anesth. 2016;30:444-448. 26847740 10.1007/s00540-016-2139-8

[28] Khalifa M. Reducing length of stay by enhancing patient’s discharge: a practical approach to improve hospital efficiency. Stud Health Technol Inform. 2017;238:157-160. 28679912

[29] Mehra T Muller CTB Volbracht J Seifert B Moos R . Predictors of high profit and high deficit outliers under SwissDRG of a tertiary care center. PLoS One. 2015;10(10):1-18. 10.1371/journal.pone.0140874 PMC4627843 26517545

[30] Holmes GM Pink GH Friedman SA. The financial performance of rural hospitals and implications for elimination of the critical access hospital program. J Rural Health. 2013;29:140-149. 23551644 10.1111/j.1748-0361.2012.00425.x

[31] SwissDRG. SwissDRG catalog. Accessed October 23, 2023. https://www.swissdrg.org/application/files/5016/7152/9703/SwissDRG-Version_12.0_Fallpauschalenkatalog_AV_2023_2023_f.pdf

[32] Presentation of the system to the Board of Directors of SwissDRG SA. SwissDRG; 2023. Accessed October 23, 2023. https://www.swissdrg.org/application/files/4016/5580/3907/Presentation_systeme_SwissDRG_Version_12.0_2023_Homepage.pdf

[33] Xu X Grossetta Nardini HK Ruger JP. Micro-costing studies in the health and medical literature: protocol for a systematic review. Syst Rev. 2014;3:47. 24887208 10.1186/2046-4053-3-47 PMC4036677

[34] Dwivedi AK Mallawaarachchi I Alvarado LA. Analysis of small sample size studies using nonparametric bootstrap test with pooled resampling method. Stat Med. 2017;36:2187-2205. 28276584 10.1002/sim.7263

[35] Vladeck BC. Medicare hospital payment by diagnosis-related groups. Ann Intern Med. 1984;100:576. 6422818 10.7326/0003-4819-100-4-576

[36] Jentzsch T Seifert B Neuhaus V Moos RM. Predictors for shorter and longer length of hospital stay outliers: a retrospective case-control study of 8247 patients at a university hospital trauma department. Swiss Med Wkly. 2018;1482018;148:w14650. 10.4414/smw.2018.14650 30141523

[37] McMahon LF Shapiro LR Weissfeld LA. Prior hospitalization experience of DRG outliers versus inliers. Med Care. 1988;26:423-429. 3127642 10.1097/00005650-198804000-00011

[38] Dowling WL. Prospective reimbursement of hospitals. Inquiry. 1974;11:163-180. 4278236

[39] Whetsell GW. The history and evolution of hospital payment systems: how did we get here? Nurs Adm Q. 1999;23:1-15. 10711138

[40] Hsiao WC Sapolsky HM Dunn DL Weiner SL. Lessons of the new jersey DRG payment system. Health Aff. 1986;5:32-45. 10.1377/hlthaff.5.2.32 3091466

[41] SwissDRG. Notions élémentaires: Borne de durée de séjour et outliers. SwissDRG. Accessed April 20, 2023. https://www.swissdrg.org/fr/portrait/communication/notions-elementaires

[42] Flynn BC de Perio M Hughes E Silvay G. The need for specialized preanesthesia clinics for day admission cardiac and major vascular surgery patients. Semin Cardiothorac Vasc Anesth. 2009;13:241-248. 19945957 10.1177/1089253209352252

[43] Bernard AC Summers A Thomas J , et al. Novel Spanish translators for acute care nurses and physicians: usefulness and effect on practitioner’s stress. Am J Crit Care. 2005;14:545-550. 16249591

